# Effect of Phenolic Resin on the Rheological and Morphological Characteristics of Styrene-Butadiene Rubber-Modified Asphalt

**DOI:** 10.3390/ma13245836

**Published:** 2020-12-21

**Authors:** Peifeng Cheng, Yiming Li, Zhanming Zhang

**Affiliations:** Department of Civil Engineering, Northeast Forestry University, Harbin 150036, China; chengpeifeng@nefu.edu.cn (P.C.); zhanming@nefu.edu.cn (Z.Z.)

**Keywords:** phenolic resin, SBR, rheological, morphological, thermal aging

## Abstract

To improve the thermal-aging stability and rheological performance of styrene–butadiene rubber (SBR)-modified asphalt, phenolic resin (PF) was introduced in the process of preparing SBR-modified asphalt by melt blending. The effect of PF and SBR on the high and low-temperature rheological performance of the asphalt binder before and after aging was evaluated by a temperature and frequency sweep using a dynamic shear rheometer (DSR). Fourier transform infrared spectroscopy (FTIR), gel permeation chromatography (GPC), and fluorescence microscopy (FM) were used to further investigate the effect of PF and SBR on the thermal stability and morphological characteristics of the asphalt binder. The results showed that the addition of PF can enhance the high-temperature deformation resistance and short-term aging resistance of SBR-modified asphalt. Moreover, PF and SBR form an embedded network structure within the asphalt binder and alleviate the deterioration of the polymer during the aging process. Compared with SBR-modified asphalt, the chemical system of composite-modified asphalt is more stable, and it can remain stable with an aging time of less than 5 h.

## 1. Introduction

With the rapid increase of traffic loading and frequency, the base asphalt can hardly meet the requirements of both high- and low-temperature performance simultaneously [1,2]. Fortunately, research has found that high-performance asphalt binders can be obtained by the incorporation of polymers through a mechanical mixing or chemical reaction method [3]. Currently, various polymers have been used to modify the base asphalt, such as styrene–butadiene-styrene (SBS), styrene–butadiene rubber (SBR), and polyethylene (PE) to improve the performance of road pavement [4]. In addition, research shown that the content and type of the polymers in asphalt have a significant impact on the durability of asphalt pavements [5,6,7]. Zhu compared the effects of polymer modified asphalt (SBS content 3%) and higher polymer (HP) content (SBS content 7.5%) on improving the long-term aging resistance of asphalt binders. The results showed that the HP asphalt binder has better long-term aging resistance compared with polymer modified asphalt binder (PMB) [8]. Moreover, it should notice that as polymer content increases, phase inversion or phase separate may occur in some PMBs [9]. H.I. Al-Abdul Wahhab et al. studied the effect of various recycled polyethylene (RHDPE & RLDPE) and polypropylene (RPP) in combination with SBS and polybilt (PB) on the storage stability, recovering tendency, and high temperature performance of asphalt. They found that all the RPWs yield modified asphalt with improved high-temperature performance, besides, the content of RHDPE, RLDPE, and RPP with SBS will influence the storage stability of the modified asphalt [10].

Of these, SBR as a rubber modifier can significantly improve the stress relaxation ability of asphalt binders at low temperatures [11]. In 1987, an Engineering Brief from the U.S. Federal Highway Administration described the advantages of SBR-modified asphalt for improving the performance of asphalt pavements [12]. Moreover, according to Becker et al. [13], SBR latex polymers increase the ductility of asphalt binders, which allows the pavement to have better crack resistance at low temperatures, as found by the Florida Department of Transportation (FDOT). Thus, the implementation of SBR-modified asphalt in cold regions has many unique advantages compared with other modifiers [14]. Unfortunately, some defects of SBR-modified asphalt have also been found during field implementation; for example, the rutting resistance of SBR-modified asphalt is not always sufficient, and its compatibility is poor [15]. Furthermore, some researchers have found that, due to the special molecular structure of SBR, it is much easier to oxidize, and the flexibility of SBR-modified asphalt at low temperatures deteriorates after short-term aging [16]. These defects limit SBR-modified asphalt’s further application and development. Thus, extensive research has been conducted to obtain a high-quality SBR-modified asphalt [17,18,19,20]. 

Phenolic resin (PF), as one of the earliest synthetic resin materials, has a relatively low price and good deformation resistance [21]. Therefore, PF has often been used as a rubber reinforcement and curing agent [22,23]. Derakhshandeh [24] investigated the thermal stability, mechanical properties and microstructure of NBR and SBR blended with PF, respectively. The results showed that the addition of PF affected the cross-linking density of the rubber phase and played a leading role in the curing process of rubber. Moreover, partial PF particles were embedded in the rubber phase. Shojaei and Faghihi [25] found that organoclay (OC) and PF can accelerate the curing rate of SBR, increase the cross-linking density of the rubber phase and improve the thermal stability of SBR. Mirabedini et al. [26] studied the effect of PF content on the viscoelastic behavior and thermal stability of the NBR/PF blend. The results showed that the addition of PF forms an interpenetrating network structure between rubber and resin in the blend. With the increase in PF content, the crosslink density of the blend also increases; thus, the blends show more elastic behavior and better deformation resistance. Moreover, in our previous research, we found that after combining 4% of SBR with 3% of PF, the temperature sensitivity of the SBR-modified asphalt binder is significantly decreased, and the high-temperature performance and moisture resistance of SBR-modified asphalt mixture is improved [27]. However, the effects of PF on the thermal aging resistance, rheological characteristics, and morphological and modification mechanisms have not been well studied. 

In recent years, several methods have been used to analyze the chemical and morphological structure of asphalt binders, such as gel permeation chromatography (GPC), Fourier transform infrared spectroscopy (FTIR) and fluorescence microscopy (FM). Research has shown that FTIR, GPC and FM can effectively describe and analyze the modification mechanism and microstructure characteristics of polymer-modified asphalt [28,29,30,31]. Therefore, FTIR, GPC and FM were selected to investigate the effect of aging and the modification mechanism of asphalt binders at the microscopic level.

The main objective of this study was to investigate the effect of PF on the thermal stability and rheological performance of SBR-modified asphalt and analyze its morphological evolution at different aging degrees. The rheological properties of base asphalt (A9), SBR-modified asphalt (S4) and SBR-modified asphalt with PF (S4P3) were evaluated by the frequency sweep test and temperature sweep test, respectively. During the aging process, the morphological evolution of asphalt binders was investigated by FTIR, GPC and FM.

## 2. Materials and Methods 

### 2.1. Materials

The asphalt binder used in this study was an 80/100 penetration graded material provided by the Panjin Asphalt Factory. The physical properties of the base asphalt are shown in Table 1. The star-like SBR modifier was produced by Zibo Petrochemical Co. Ltd (Zibo, China). with 22.3–24.6 wt% of styrene content. The phenolic resin was provided by Zhenzhou Resin Factory. Table 2 presents the pertinent physical properties of PF.

### 2.2. Methods

#### 2.2.1. Asphalt Binder Preparation

First, the base asphalt was heated to a molten state. Second, 4% of SBR (by weight of asphalt) was added under 800 rpm at 155 °C for 15 min; then, keep the temperature constantly and a high shear mixer with a speed of 3000 rpm was used for 30 min to fully disperse the polymer in the asphalt binder. Finally, 3% of PF (by weight of asphalt) was added and sheared together at 165 °C for 30 min at a speed of 3500 rpm.

#### 2.2.2. Rheological Measurements 

The rheological characteristic of asphalt binders was determined with an AntonPaar modular compact rheometer (MCR 302, Shanghai, China). The frequency sweep was performed with controlled strain at 1%, and the loading frequency was varied in a logarithmic manner from 0.1 to 60 Hz at temperatures ranging from −20 °C to 60 °C (with an interval of 10 °C) to sweep the sample before and after short-term aging. Two different diameter parallel plates were used for testing: 8 mm diameter with a 2 mm gap at test temperatures lower than 30 °C and 25 mm diameter with a 1-mm gap at test temperatures higher than 30 °C. 

The complex modulus ($G^{*}$) and phase angle (δ) were collected to analyze the rheological performance and construct master curves. With 30 °C as the reference temperature, a master curve was constructed based on the principle of time-temperature superposition (TTS). The shift factors were determined using the Williams–Landel–Ferry (WLF) equation, as shown in Equation (1). The modified Christensen–Anderson–Marasteanu (CAM) model, defined by Equations (2) and (3), was used to fit the shifted complex shear modulus and phase angle, respectively [32].

(1) $$\log a_{T}=\frac{-c_{1}(T-T_{r})}{c_{2}+(T-T_{r})},$$

(2) $$\left|G^{*}\right|=\frac{G_{g}^{*}}{{\left[1+{\left(f_{c}/f^{\prime }\right)}^{k}\right]}^{m_{c}/k}},$$

(3) $$\delta =90I-(90I-\delta_{m}){\left[1+(\frac{\log(f_{d}/f^{\prime })}{R_{d}})\right]}^{-m_{d}/2}.$$

where, $a_{T}$ is the horizontal shift factor at the temperature T; T is the test temperature, °C; $T_{r}$ is the reference temperature, °C; $\left|G^{*}\right|$ is the dynamic modulus, MPa; $f^{\prime }$ is he reduced frequency, Hz; $G_{g}^{*}$ is the glassy modulus, MPa; $f_{c}$, $f_{d}$ is the location parameter; δ is the phase angle, °; I is the dummy variable (if $f^{\prime }$>$f_{d}$, I = 0; or I = 1); and $m_{c},k,R_{d},m_{d}$ are the shape parameters. 

The temperature sweep test was conducted with controlled strain at 0.1% and 1% for low-temperature (from −20 to 30 °C) and high-temperature sweeps (from 30 to 80 °C), respectively. The loading frequency was 10 rad/s, and the type of parallel plate used was consistent with the frequency sweep test.

#### 2.2.3. Aging Method 

Asphalt binders were aged by using the following two different aging protocols: short-term aging was performed by the rolling thin film oven test (RTFOT) at 163 °C for 85 min according to American Society for Testing and Materials (ASTM) D 2872-04. Different aging degrees can be simulated through the extend RTFOT test [33]. To investigate the effect of different aging extents, all the binders were aged by RTFOT at 163 °C with airflow of 4 L/min for 1, 3, 5 and 7 h. 

#### 2.2.4. Fourier-Transform Infrared Spectroscopy (FTIR)

A Nicolet is 50 FTIR was adopted to analyze the evolution of the chemical bonds of asphalt binders. Wavelengths ranging from 4000 cm^−1^ to 400 cm^−1^ were employed. The binder was dissolved in solvent (carbon disulfide) with a 5 wt % concentration, then dropped onto KBr plates for FTIR analysis.

#### 2.2.5. Gel Permeation Chromatography (GPC)

GPC was performed using the equipment model of an Agilent 1100 with computerized software for the chromatographic analysis of binders. The columns were kept at 30 °C throughout the test in a column oven. Before the GPC analysis, all binders were dissolved into tetrahydrofuran (THF). The mobile phase was THF at a flow rate of 1.0 mL/min. The concentration of samples used was 1.0–2.0 g/L.

#### 2.2.6. Fluorescence Microscope (FM)

An AxioImager A2 optical microscope (Zeiss, Oberkochen, Germany). was used to observe the morphology of asphalt binders. Small amounts of heated samples were placed between microscope slides, and then the samples were viewed under a microscope at a magnification of 400.

## 3. Results and Discussion

### 3.1. Frequency Sweep Tests

**Figure 1:**
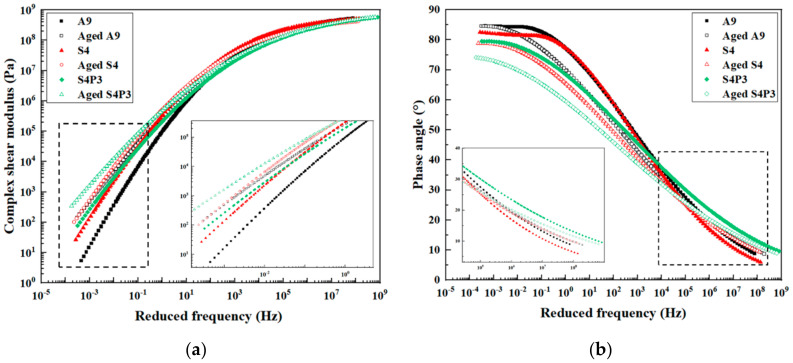
Master curves of asphalt binders before and after aging. (**a**) Complex shear modulus; (**b**) phase angle.

The complex shear modulus master curves for A9, S4 and S4P3 before and after aging are shown in Figure 1a. In general, the complex shear modulus decreased as the reduced frequency increased; besides this, the differences in all the binders gradually reduced when the frequency was around 10^3^ to 10^9^ Hz, and the shear modulus of all asphalt binders was finally around 10^9^ Pa. According to the time-temperature superposition (TTS) principle, the higher the frequency response, the lower the temperature. This means that as the temperature decreased, the deformation resistance of the asphalt binders increased and the binders showed more elastic behavior. At lower frequencies (higher temperatures), A9 had the lowest complex shear modulus and S4 had the higher complex shear modulus. This indicates that, after adding the SBR, the high-temperature performance of the asphalt binder increased, and the complex shear modulus was further increased after adding PF; S4P3 exhibited the highest value of complex shear modulus. We also found that in the higher-frequency region (lower temperatures), the difference in the complex shear moduli of S4P3 and S4 was not significant. This indicated that S4P3 has a better high-temperature performance than A9 and S4, and there may be a slight effect of low temperature after adding PF. This will be further discussed in the temperature sweep. Overall, compared with the unaged asphalt binder, aging further increased the complex shear modulus, indicating that aging reduces flexibility. In addition, A9 had the highest complex shear modulus difference after aging, while the difference between S4 and S4P3 was lower. This shows that the aging resistance of the asphalt binder was enhanced after adding SBR and PF.

The phase angle master curves for A9, S4 and S4P3 before and after aging are shown in Figure 1b. The phase angle can be used as an indicator of the viscoelastic behavior of asphalt binders; besides this, researchers have also found that the temperature of the phase angle at around 30° can effectively predict the low-temperature performance [34]. Figure 1b shows that with increasing frequency, the phase angle gradually decreased, which means that as the temperature decreased, the elasticity of the asphalt binder gradually increased. Moreover, S4P3 showed the lowest phase angle value compared with S4 and A9 in a higher temperature region, and the difference in S4 and A9 was not obvious when the frequency was around 10^−1^ to 10^2^ Hz. A lower phase angle shows a higher elastic response; this therefore indicates that SBR resulted in a limited improvement on the high-temperature performance of asphalt binders, while PF could significantly improve the high-temperature performance of asphalt binders. Moreover, compared with other binders, S4P3 had a higher phase angle value in the high-frequency region (around 10^5^ to 10^9^ Hz), which means that S4P3 was more flexible under the same conditions.

### 3.2. Temperature Sweep Tests

**Figure 2:**
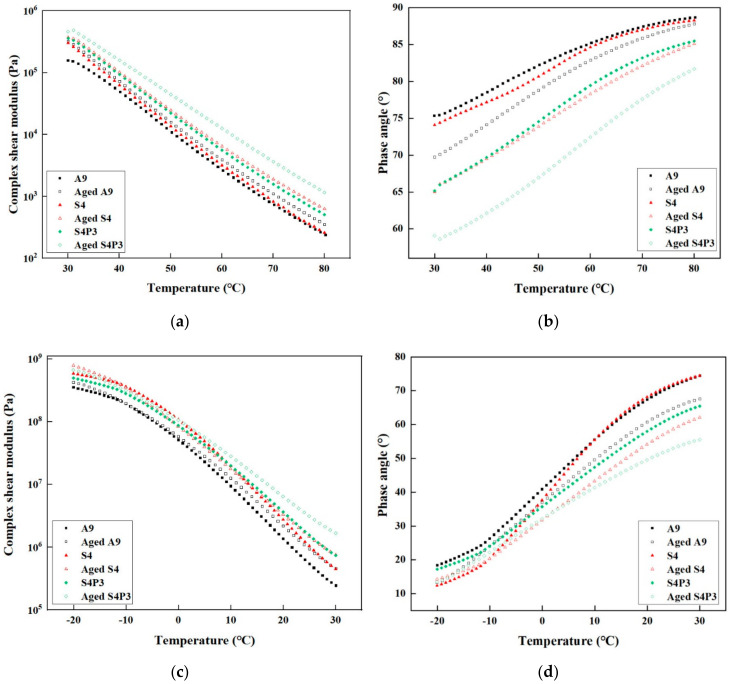
Rheological properties of asphalt binders before and after aging. (**a**,**b**) From 30 to 80 °C; (**c**,**d**) from −20 to 30 °C.

To investigate the rheological properties of A9, S4 and S4P3 before and after aging under a certain temperature range (−20 to 80 °C), temperature sweep tests were performed. The test results are shown in Figure 2a–d. As shown in Figure 2a,b, S4P3 had the highest complex shear modulus and lowest phase angle value over the whole temperature range, which shows that S4P3 has a better high-temperature performance. Moreover, it was observed that there was no obvious difference in the complex shear modulus and phase angle between A9 and S4 when the sweep ranged from 60 °C to 80 °C. This indicates that in the high-temperature aspect, the improvement caused by SBR was limited.

For low-temperature sweep results are shown in Figure 2c,d. Obtained results show that as the temperature decreased, the complex shear modulus increased while the phase angle decreased. As shown in Figure 2c,d, S4 had the highest value of the complex shear modulus and lowest value of the phase angle when the temperature was lower than 0 °C. Moreover, when the sweep temperature was lower than −10 °C, the complex shear modulus and phase angle of the three asphalt binders tended to be stable. This means that the asphalt binders tended to be elastomers at lower temperatures, and the change in temperature had a slight effect on the modulus. Compared with S4, when the temperature was lower than −10 °C, the complex shear modulus of S4P3 was slightly lower than that of S4. 

Generally, binders with higher complex modulus crack more quickly as these binders are unable to relax thermal stresses at low temperatures [3]. Thus, S4P3 has better deformation ability at lower temperatures. In addition, Figure 2d shows the slope of the phase angle of S4 and A9 changes steeper when the temperature exceeded −10 °C, while the change of S4P3 is slight. In general, the curvature of the phase angle can reflect the sensitivity of the material to temperature. Hence, it also means that S4P3 has a lower susceptibility to temperature. This is mainly because the embedded network structure formed by PF and SBR reduces the impact of temperature changes on the performance of asphalt and rubber particles. Furthermore, these embedded PF particles do not inhibit the deformation and recovery of rubber under the action of external stress, so S4P3 has better performance at lower temperatures.

### 3.3. Aging Index (AI)

To quantitatively analyze the effect of different aging degrees on the rheological properties of asphalt binders, AI was used to evaluate the aging degree, calculated by Equation (4). High values of this ratio indicate a relatively large degree of binder hardening [35,36]. The G*/sin (*δ*) was collected by the temperature sweep test with a controlled strain at 10%, and the loading frequency was 10 rad/s.

(4) $$AI=\left|\frac{G*/\sin{\left(\delta \right)}_{Aged}}{G*/\sin{\left(\delta \right)}_{Unaged}}\right|.$$

where G*/sin (*δ*)*_Aged_* is the asphalt binder rutting parameter values obtained for the aged material and G*/sin (*δ*)*_Unaged_* is the asphalt binder rutting parameter values obtained for the unaged material.

**Figure 3:**
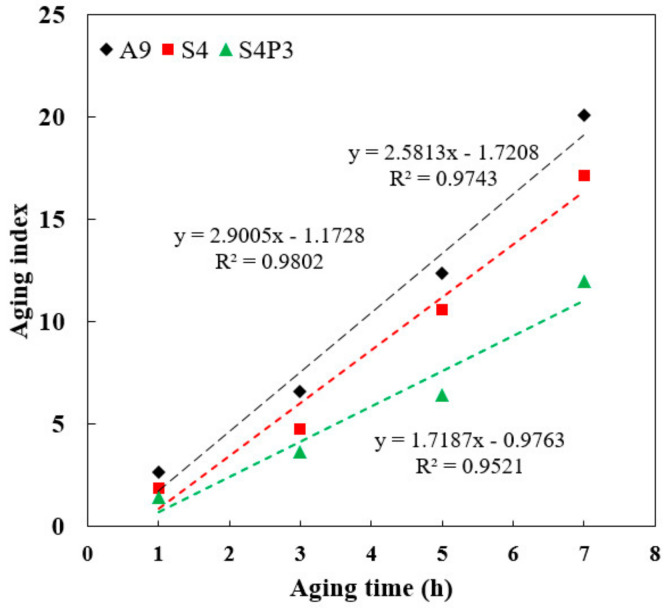
Correlation diagram of aging time with AI.

The results are shown in Figure 3 and Table 3 and show that the AI increased as the aging time increased for all the binders. Furthermore, as the aging time increased, the degree of aging increased in a linear fashion. Moreover, A9 had the highest AI value and slope, and S4P3 had the lowest value. When the aging time was changed from one hour to three hours, the AI index of S4 and S4P3 increased by 157% and 86%, respectively. This indicated that S4P3 has better aging resistance than S4, and the aging time has less influence on its rheological properties. This is mainly because a stable cross-linking system was formed by PF and SBR, which inhibited the volatilization of light components, and thus the aging resistance of S4P3 was improved. However, it should be noted that the difference between S4 and S4P3 was not significant for a short period of aging. As the aging time increased, the difference in the AI between the S4P3 and S4 also gradually increased, and this difference was obvious when the aging time was 7 h. This indicated that, in the initial aging stage, due to the swelling and adsorption of rubber particles in the asphalt, the aging resistance of the asphalt binder was improved to a certain extent, but with the increase in the aging time and aging degree deepening, the rubber particles also aged and disintegrated. Thus, the aging resistance of asphalt gradually weakened.

### 3.4. FTIR Analysis

**Figure 4:**
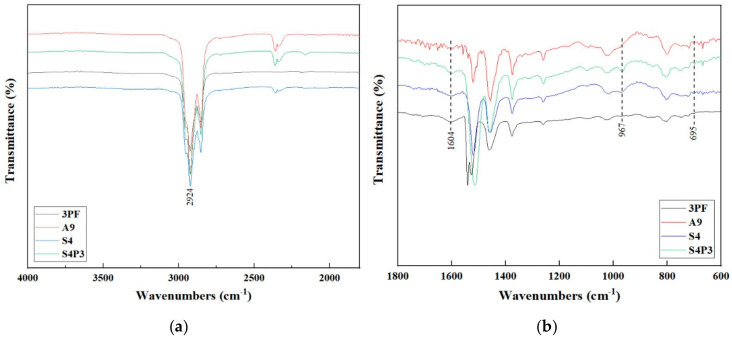
FTIR spectra of asphalt binder before aging. (**a**) 4000 to 1500 cm^−1^; (**b**) 1800 to 600 cm^−1^.

**Figure 5:**
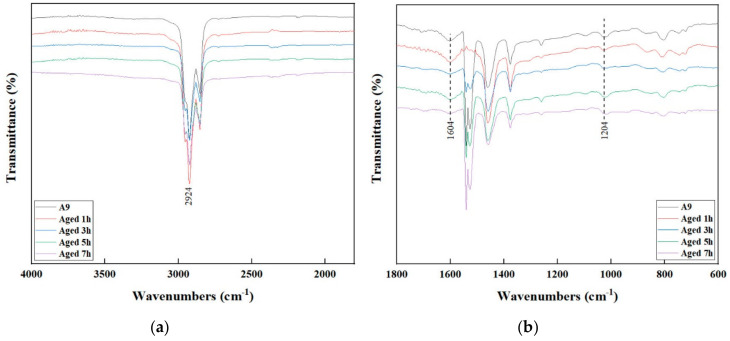
FTIR spectra of A9 at different aging times. (**a**) 4000 to 1500 cm^−1^; (**b**) 1800 to 600 cm^−1^.

**Figure 6:**
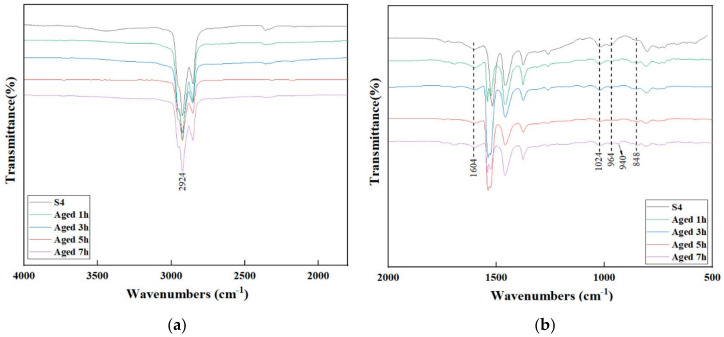
FTIR spectra of S4 at different aging times. (**a**) 4000 to 1500 cm^−1^; (**b**) 1800 to 600 cm^−1^.

**Figure 7:**
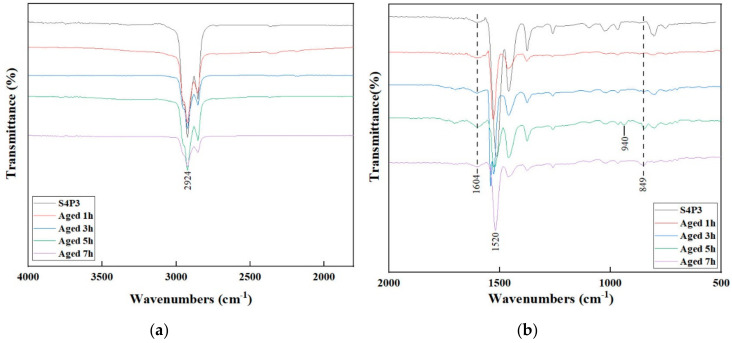
FTIR spectra of S4P3 at different aging times. (**a**) 4000 to 1500 cm^−1^; (**b**) 1800 to 600 cm^−1^.

The characteristic peak assignment of the spectra for the binders is shown in Table 4. To investigate the PF modification mechanism, a FTIR test was also conducted on pure PF-modified asphalt (3PF). The FTIR spectra of A9, S4 and S4P3 before and after aging are given in Figure 4, Figure 5, Figure 6 and Figure 7, respectively. 

The FTIR spectra of A9, S4, 3PF and S4P3 are shown in Figure 4. At 967 cm^−1^, S4 exhibited a new absorption peak due to the SBR characteristic peak vibration. Moreover, no new peak was generated compared with the A9 spectrum. This indicated that the modification mechanism of SBR was mainly physical modification without a chemical reaction. Compared with 3PF and S4, new absorption peaks appeared at 967 cm^−1^ and 695 cm^−1^, and the intensity of the absorption peak at 1515 cm^−1^ increased significantly. This indicated that the functional groups in the asphalt were transformed after adding PF, and more stable C-C bonds were generated. This also means that the modification mechanism of PSBR is a physical modification and is accompanied by chemical reactions.

The FTIR spectrum of A9 at different aging times is shown in Figure 5. During the whole aging process, only the intensity of the absorption peaks changed significantly. This indicated there were no functional group changes and generations, but some component transfers were present. Especially for the absorption peaks at 1604 cm^−1^ and 1024 cm^−1^, their intensity changed significantly after aging for one hour. This showed that the chemical system of A9 is more sensitive to aging, and more carbonyl groups and sulfoxides will be formed in the early stage of aging.

Figure 6 shows the chemical bond changes in S4 at different aging times. After one hour of aging, a new absorption peak appeared at 849 cm^−1^, and the intensity of the absorption peak also increased as the aging time increased. Moreover, when the aging time was 7 h the intensity increase was most obvious and a new absorption peak appeared at 940 cm^−1^. This indicated that S4 was more sensitive to aging, and chemical reactions occurred in the initial stage of aging, so some chemical bonds were broken and new chemical bonds were formed. This is mainly because a higher number of butadiene structures contained in the SBR molecule makes it easier for it to be oxidized or decomposed in short-term aging.

The FTIR spectrum of S4P3 is shown in Figure 7. Before 5 h of aging, there were no significant changes in the absorption peaks; the strength changed as the aging time increased. This indicated that, before 5 h of aging, S4P3 could keep the chemical system stable with better thermal aging resistance than S4. When the aging time was 5 h, the absorption peaks changed clearly at 940 cm^−1^ and 849 cm^−1^. In addition, with the aging time increasing to 7 h, the intensity of the absorption peak at 940 cm^−1^ and 1520 cm^−1^ increased significantly, and the absorption peak at 848 cm^−1^ was broadens.

It indicated that when the aging time exceeds 5 h, the chemical system of S4P3 became non-stable, and these peaks transferred to another functional group. Thus, S4P3 has good thermal aging resistance within 5 h of aging; after that, with the aging time increasing, the thermal aging resistance of S4P3 also degrades, which lead to make the chemical bond became non-stable.

**Figure 8:**
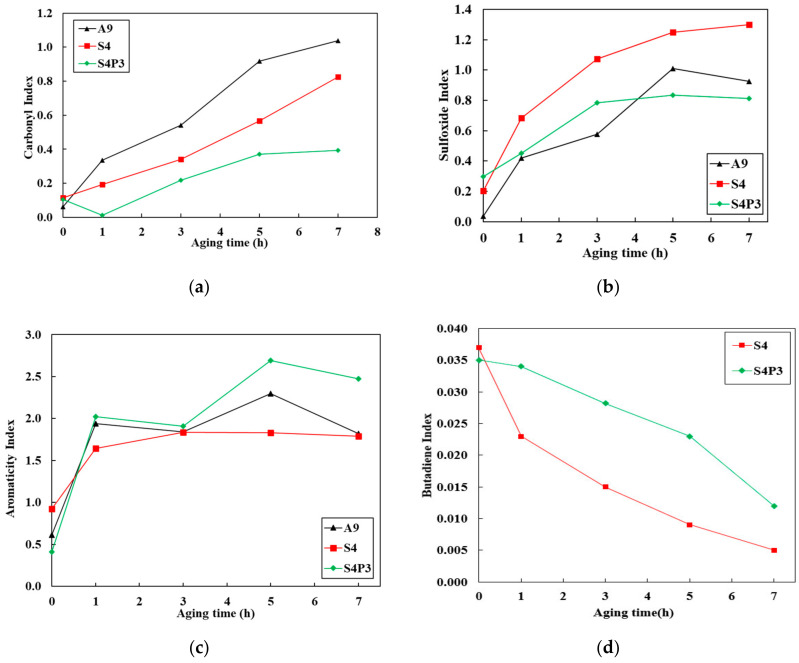
Structure index evolution at different aging times. (**a**) Carbonyl index; (**b**) sulfoxide index; (**c**) aromaticity index; (**b**) butadiene index.

To quantitatively analyze the functional group evolution of asphalt binders at different aging degrees, the structural indices *I_C=O_*, *I_S=O_*, *I_C=C_*, and polymer index (*I_BI_*) were used [37,38]. The *I_C=O_*, *I_S=O_*, *I_C=C_*, and *I_BI_* were calculated by using Equations (5)–(8), respectively, as presented in Figure 8a–d.

(5) $$I_{C=O}=\frac{Area\;of\;the\;carbonyl\;around\;1705\;cm^{-1}\;}{Area\;of\;the\;CH_{3}\;stretching\;around\;2926\;cm^{-1}},$$

(6) $$I_{S=O}=\frac{Area\;of\;the\;sulfoxide\;around\;1024\;cm^{-1}\;}{Area\;of\;the\;CH_{3}\;stretching\;around\;2926\;cm^{-1}},$$

(7) $$I_{C=C}=\frac{Area\;of\;the\;aromatics\;around\;1604\;cm^{-1}\;}{Area\;of\;the\;CH_{3}\;stretching\;around\;2926\;cm^{-1}},$$

(8) $$I_{BI}=\frac{Area\;of\;the\;butadiene\;around\;964\;cm^{-1}\;}{Area\;of\;the\;CH_{3}\;stretching\;around\;2926\;cm^{-1}},$$

Figure 8a,b show the carbonyl and sulfoxide evolution at the different aging times, respectively. In general, as the aging time increased, the carbonyl and sulfoxide rapidly increased, except the carbonyl of S4P3 at the initial stage decreased from unaged to 1 h of aging. Moreover, it was found that A9 had the highest rate of formation of carbonyl compounds, which was less in S4 and S4P3 when compared with A9; besides this, S4P3 had the lowest rate compared with other binders. This means that aging has a significant effect on A9, and as the aging time increases, the chemical reaction gradually increases. Moreover, the thermal aging resistance of A9 increased after being composited with SBR, but this improvement was limited and S4P3 had better thermal aging resistance compared with S4. It is well known that the better dispersibility of asphaltenes leads to increased formation of carbonyl compounds [39]. However, after the addition of PF and SBR, the asphaltene network of the base asphalt was disturbed; thus, carbonyl formation was reduced. A clear increase in sulfoxide was observed for all binders; S4 had a higher slope compared with A9 and S4P3. These increases could be due to the reaction of oxygen with the perhydroaromatic ring to form hydroperoxides, and thus ketones and sulfoxides were formed. Moreover, after 7 h of aging, the sulfoxide compounds of A9 were reduced. This is attributed to the decomposition of sulfoxides after prolonged periods of aging [40,41]. 

Figure 8c shows the aromatic index evolution for different aging times. We can observe that the evolution of the aromatic index for all the asphalt binders was greatly different, especially for S4 compared with A9 and S4P3. In the initial aging stage, the aromatic fraction increased for both asphalt binders. This increase is certainly the result of the aromatization of the naphthenic compounds [42]. After one hour of aging, the increase rate of the aromatic fraction of S4 was decreased; on the contrary, the aromatic compounds of A9 and S4P3 were decreased. The increase rate of aromatics of A9 and S4P3 rapidly increased from 3 h to 5 h. The same phenomenon was also found by Nivitha [39]. Therefore, during the aging process, the addition of modifiers will cause different changes in the composition of asphalt, and this change will vary greatly due to the different modifiers, especially for the aromatic fraction. In summary, it is reasonable and accurate to use the carbonyl index to evaluate the aging effect of asphalt. Figure 8d shows that, with increasing aging time, the *I_BI_* of S4 and S4P3 both decrease. The *I_BI_* of S4 changes significantly in the early stage of aging, while the change in S4P3 is relatively small. The *I_BI_* in asphalt can reflect the change in the rubber particles. This means that rubber also deteriorates during the aging process, and the addition of PF reduces the degradation of the rubber during the aging process.

### 3.5. GPC Analysis 

**Figure 9:**
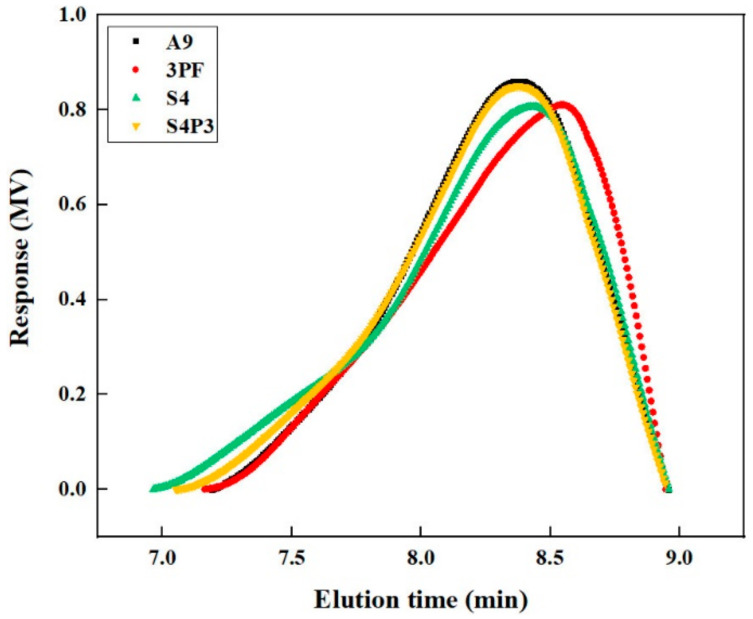
Gel permeation chromatography (GPC) of asphalt before aging.

The molecular weight and molecular weight distribution of the asphalt are the inherent reasons for the performance of the asphalt, and thus have a great impact on its performance. Therefore, in this study, GPC was conducted to investigate the evolution of the molecular weight of the asphalt binders during the aging process to analyze the aging effect [43,44]. Figure 9 and Table 5 shows the GPC results of asphalt binders before aging. We found that 3PF is mainly composed of small molecules (SMS) and medium molecules (MMS), as we expected. Compared with S4, the contents of large molecules (LMS) and SMS of S4P3 were increased. This is due to the entanglement and cross-linking between PF and rubber, which increased the overall average relative molecular mass. Our research found that the content of LMS in asphalt has a good correlation with the high and low-temperature performance of asphalt [45]. Therefore, PF improves the high-temperature performance while maintaining better low-temperature performance, which also verified the results of the temperature sweep test results.

**Figure 10:**
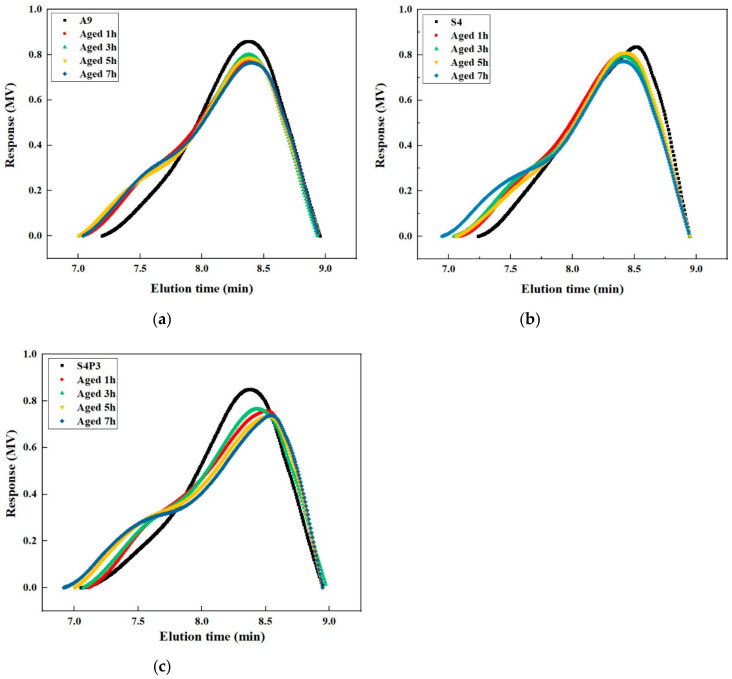
GPC evolution of asphalt binders at different aging times. (**a**) A9; (**b**) S4; (**c**) S4P3.

Figure 10a and Table 6 show the molecular weight distribution evolution of A9 at different aging times. In general, as the aging time increased, the MMS were converted to LMS and SMS during the aging process, and the content of LMS and SMS increased gradually. It is worth noting that, when the aging time was 7 h, the LMS decreased compared with 5 h of aging. This may be due to excessive aging, which caused the LMS to decompose.

Figure 10b and Table 6 shows the molecular weight distribution evolution of S4 at different aging times. As the aging time increased, the SMS and MMS were gradually converted to larger molecules. After one hour of aging, the content of LMS in the S4 increased significantly. This was due to the continued swelling of the rubber particles increasing the cross-link density and increasing the average molecular weight. Compared with 1 h of aging, the LMS in S4 was decreased after 3 h aging. This may be due to the rubber particle degradation into SMS under the influence of aging. It also means that S4 has poor aging resistance. Even when subjected to short-term aging, significant oxidation reactions occurred inside. When the aging time exceeded 5 h, the LMS content of the asphalt increased again. This was due to the deepening of the aging degree and the polymerization of more molecules in the asphalt.

The molecular weight distribution evolution of S4P3 at different aging times is shown in Figure 10c and Table 6. S4P3 shows significantly different changes in the molecular weight distribution evolution compared with S4. In general, during the whole aging process, the changes in the MMS in the S4P3 asphalt are more obvious, and the MMS are converted into LMS and SMS. Thus, the content of LMS and SMS in S4P3 was increased. The increase in LMS and SMS also contributed to the improvement of the performance of S4P3. Moreover, when the aging time was less than 5 h, there was no obvious degradation of the rubber particles, which also shows that the addition of PF can improve the thermal aging stability of asphalt binders.

### 3.6. Morphology Evolution 

**Figure 11:**
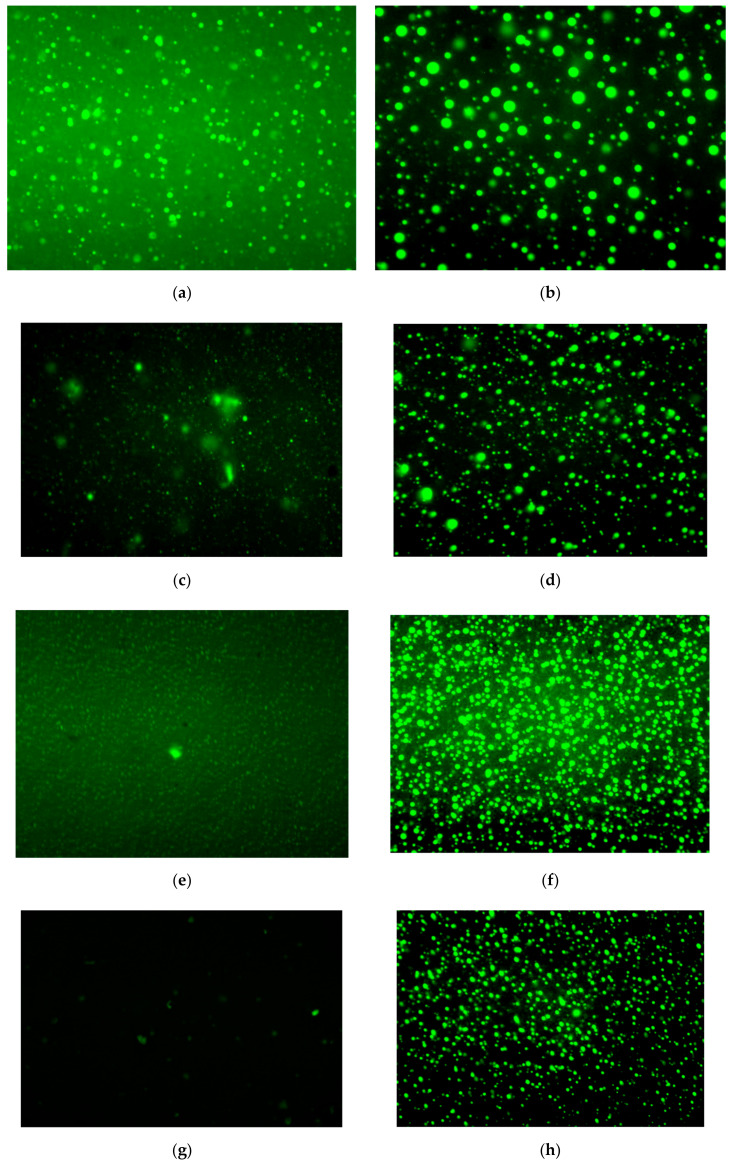

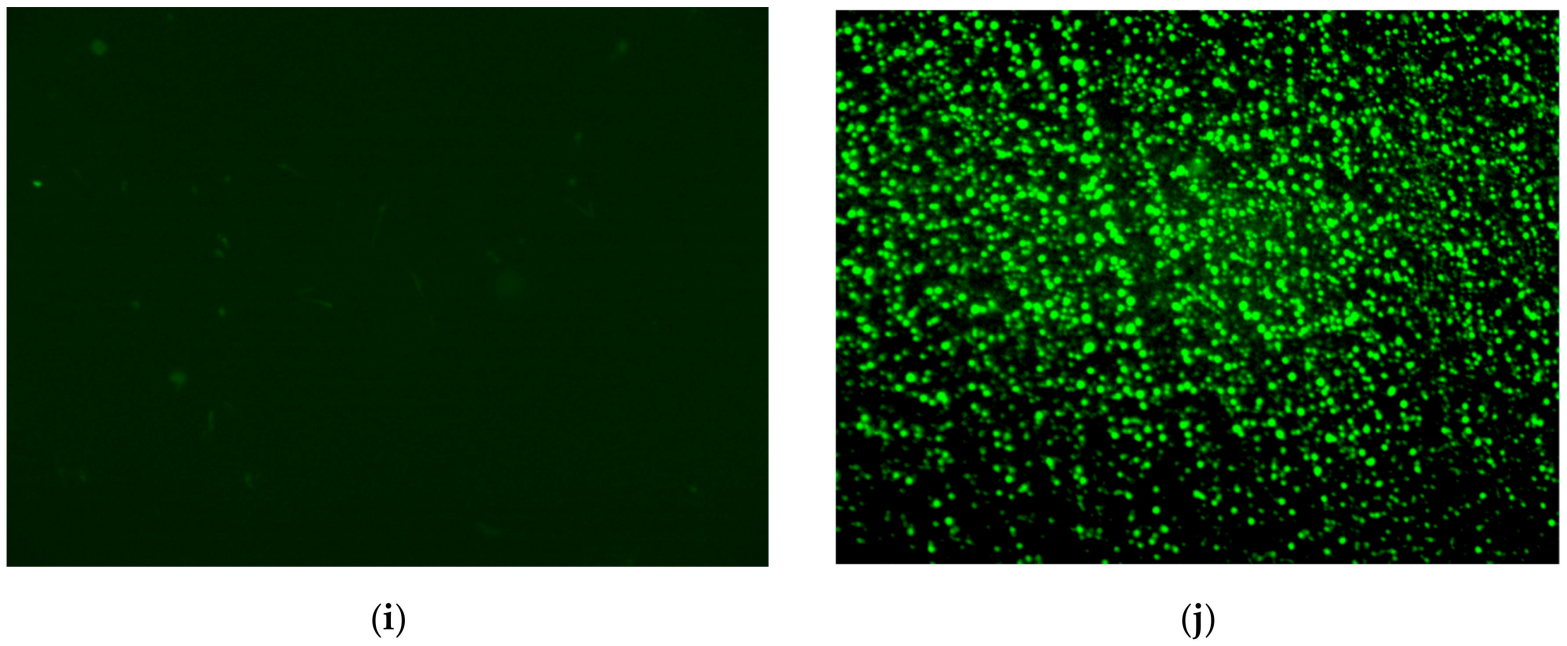
Microstructure evolution of modified asphalt under different aging degrees. (**a**) Virgin S4; (**b**) Virgin S4P3; (**c**) S4 aged for 1 h; (**b**) S4P3 aged for 1 h; (**e**) S4 aged for 3 h; (**f**) S4P3 aged for 3 h; (**g**) S4 aged for 5 h; (**h**) S4P3 aged for 5 h; (**i**) S4 aged for 7 h; (**j**) S4P3 aged for 7 h.

Asphalt and polymers reflect different colors under different light sources under a fluorescence microscope. Therefore, the dispersion state and particle size of the polymer in the asphalt can be observed. The morphological structures of S4 and S4P3 under different aging degrees are shown in Figure 11a–j. The brighter part is the color reflected by the polymer, and the asphalt is the darker part of the figure.

From Figure 11a,b, the size and dispersion of rubber particles in S4 and S4P3 can be seen to be roughly the same, and they have a better interfacial phase with asphalt. This indicates that the addition of PF will not affect the dispersion of SBR in asphalt and has good compatibility with asphalt. In S4P3, PF was dispersed between the asphalt’s continuous phase and rubber phase in a smaller size and formed an embedded network structure. PF showed better high-temperature performance and dimensional stability; thus, it played a key role in protection and thermal isolation, reducing the effects of temperature changes and aging on the asphalt and rubber particles.

The morphological evolution of S4 and S4P3 under different aging times is shown in Figure 11c–j. It can be seen that the size and dispersion states of the polymer in S4 and S4P3 were significantly changed as the aging time increased. Aging had the most obvious impact on S4. As the aging time increased, the size of rubber particles in S4 decreased significantly. When the aging time exceeded 3 h, the degradation of rubber particles in asphalt was the most serious. When the aging time was 7 h, the rubber phase almost completely disappeared in the asphalt. This also shows that aging has a greater impact on rubber particles, as even a short aging time will cause significant changes, which again verifies the conclusions obtained in FTIR and GPC.

Compared with S4, S4P3 showed better stability during the whole aging process, and its morphological characteristics were slightly changed. In the early stage of aging (aging for one hour), the size of rubber particles decreased slightly, and the interface strength between PF and SBR increased. As the aging time increased, the size of the rubber particles in the internal system gradually decreased, but the rate of change was relatively slow. The rubber particles still maintained a good core after aging for 3 h, PF and SBR were well dispersed in the asphalt at the transition interface and the intensity increased. When the aging time exceeded 3 h, the PF began to solidify and polymerize, changing from a well-dispersed small particle state to a larger size particle, and the size of the rubber particles also began to decrease significantly. When the aging time was 7 h, PF continued to solidify and polymerize, and obvious continuous phases were formed between the PF itself and rubber particles. Therefore, the restriction of the asphalt molecules as further strengthened, and the asphalt exhibited better resistance to deformation; however, the relative deformation ability decreased significantly.

## 4. Conclusions

Based on the obtained results, the following conclusions can be drawn:

1. PF improves the rheological properties of SBR-modified asphalt. S4P3 has good deformation resistance at high temperatures and maintains good flexibility at low temperatures.
2. The addition of PF improves the thermal aging resistance of SBR-modified asphalt and reduces the influence of aging on its performance. S4P3 has a lower degree of aging at the same aging time.
3. The FTIR and GPC results show that S4P3 has good chemical system stability and it can remain stable when the aging time is less than 5 h. Under the same aging degree, S4P3 formed fewer carbonyl and sulfoxide compounds and has a slower deterioration rate of the polymer.
4. In the S4P3 asphalt binder, PF and SBR have good compatibility with asphalt and form an embedded network structure in combination with the chemical reaction, the content of LMS in the asphalt is increased and the impact of aging and temperature on its performance is reduced.
5. As the aging time increases, the polymers will gradually decompose. After 3 h of aging, the size of the polymer in S4 is reduced, and the deterioration of the polymer is significant. After adding PF, the polymer can maintain a better core size for a longer period.

This study focused on the rheological properties and morphological characteristics of S4P3-modified asphalt binder. Thus, it is recommended for future work to study the durability of S4P3 mixture under different weather conditions and analyze its mechanism.

## Figures and Tables

**Table 1:** Physical properties of base asphalt.

Type	Physical Properties	Standard	Result
A9	Penetration (25 °C, 0.1 mm)	ASTM D5	86.4
S4			80.4
S4P3			74.1
A9	Ductility (5 °C, 5 cm/min, cm)	ASTM D113	11
S4			150
S4P3			150
A9	Softening point (°C)	ASTM D36	47.8
S4			51
S4P3			55.9
A9	Viscosity (135 °C, Pa·s)	ASTM D4402	0.68
S4			0.97
S4P3			1.17

**Table 2:** Physical properties of phenolic resin (PF).

Property	Value
Moisture (%)	≤2.0
Softening point (°C)	98–110
Fluidity (mm)	20–40
Polymerization speeds (150 °C)	49~88
Free phenol (%)	2.0–4.0
Fineness% (140 mesh)	≥96

**Table 3:** Aging index (AI) at different aging times and temperatures.

Aging Time		AI					
		52 °C	58 °C	64 °C	70 °C	76 °C	Average
A9	1	2.63	2.60	2.51	2.61	2.87	2.64
	3	6.52	6.46	6.61	6.56	6.97	6.62
	5	12.51	12.42	12.42	12.33	12.27	12.39
	7	19.09	20.17	19.70	20.21	21.13	20.06
S4	1	1.89	1.87	1.87	1.90	1.84	1.87
	3	4.71	4.88	4.71	4.84	4.84	4.80
	5	10.88	10.71	10.67	10.34	10.39	10.60
	7	16.90	17.03	17.02	17.27	17.52	17.15
S4P3	1	1.48	1.44	1.47	1.41	1.49	1.46
	3	2.70	2.75	2.71	2.76	2.68	2.72
	5	5.76	5.68	5.83	5.63	5.52	5.68
	7	11.98	12.01	12.08	11.81	12.08	11.99

**Table 4:** Assignment of the main bonds in the Fourier-transform infrared (FTIR) spectrum.

Wavenumber (cm^−1^)	Assignations
2924	C-H stretching vibration
1705	C=O stretching vibration
1604	C=C in aromatics stretching vibration
1515	C-C stretching vibration
1024	S=O stretching vibration
964	Trans-C-H wagging vibration (butadiene block)
940	C-H deformation vibration of ring hydrogens
848	C-H deformation vibration
695	Ring deformation vibration

**Table 5:** Molecular weight distribution of different asphalt binders.

Size	Type			
	A9	S4	3P3	S4P3
Large molecules (LMS)	15.01%	15.21%	13.40%	15.68%
Medium molecules (MMS)	49.02%	44.06%	42.07%	44.13%
Small molecules (SMS)	35.97%	40.73%	44.53%	40.19%

**Table 6:** Molecular weight evolution of different asphalt binders.

Type	Size	Virgin	Aged 1 h	Aged 3 h	Aged 5 h	Aged 7 h
A9	LMS	15.01%	17.67%	17.73%	17.80%	17.78%
	MMS	49.02%	44.53%	43.53%	43.39%	43.33%
	SMS	35.97%	37.80%	38.74%	38.81%	38.89%
S4	LMS	15.09%	16.57%	15.68%	16.66%	17.96%
	MMS	44.06%	45.30%	43.06%	43.35%	41.93%
	SMS	40.85%	38.13%	41.26%	39.99%	40.11%
S4P3	LMS	15.68%	18.29%	18.35%	19.18%	18.24%
	MMS	44.13%	40.79%	38.52%	37.70%	36.00%
	SMS	40.19%	40.92%	43.13%	43.12%	45.76%
